# Personalising circadian hygiene educational initiatives aimed at university students—“He who has ears to hear, let him hear”

**DOI:** 10.1111/jsr.14194

**Published:** 2024-03-14

**Authors:** Gianluca Giusti, Esther D. Domenie, Lisa Zarantonello, Chiara Mangini, Paolo Ferrari, Rodolfo Costa, Sara Montagnese

**Affiliations:** ^1^ Department of Medicine University of Padova Padova Italy; ^2^ Chronobiology Section, Faculty of Health and Medical Sciences University of Surrey Guildford UK; ^3^ reMedia Web Agency S.r.l. Padova Italy; ^4^ Institute of Neuroscience, National Research Council (CNR) Padova Italy; ^5^ Department of Biomedical Sciences University of Padova Padova Italy

**Keywords:** academic performance, chronotype, circadian education, sleep, university students

## Abstract

The aim of the present study was to characterise “early drop‐outs” (*n* = 3185) out of a group of university students (*n* = 7766) engaged in an ongoing circadian education initiative, to evaluate its efficacy and direct its developments. The initiative is aimed at improving sleep timing/quality through one of two sets of circadian hygiene advice covering the timing of sleep, meals, exercise and light exposure, and it has already been shown to have a positive effect on sleep timing. This second, interim analysis confirmed the high prevalence of disturbed night sleep and social jetlag amongst students at Padova University. Three‐thousand, one‐hundred and eighty‐five (41.0%) students were early drop‐outs. These were more commonly males (46.4 versus 37.6%; *χ* ^2^ = 58, *p* < 0.0001), had later sleep–wake habits, more daytime sleepiness and worse night sleep quality. Chronotype distribution was also different, with a slight but significantly higher proportion of extremely evening/evening types amongst early drop‐outs (*χ* ^2^ = 10, *p* < 0.05). These results suggest that the more evening the student, the lower their likelihood of choosing/being able to follow circadian advice.

## 1 INTRODUCTION

Health‐related surveys and educational initiatives, especially when advertised and/or provided online, suffer from multiple types of selection bias, to include a personal interest in the object of the initiative, computer/social media literacy, and other factors. Unless the features of the population to which the initiative is aimed at are known in detail beforehand, it is difficult to evaluate participation and attrition in a meaningful fashion post hoc. Here we make use of a group of “early drop‐outs”, and of initial, self‐reported compliance with the circadian education initiative SleepRhythm Unipd (fully described in our first, published paper on the initiative: Montagnese et al., 2022) to evaluate some aspects of its efficacy and direct its developments. In brief, the initiative—which started in October 2019, is ongoing and described in detail in Montagnese et al. (2022)—is aimed at improving sleep timing and sleep quality through one of two sets of circadian hygiene advice, covering the timing of sleep, meals, physical exercise and light exposure.

## 2 METHODS

All methods used in this study are described in full detail in Montagnese et al. (2022). All active students at the University of Padova were offered the possibility to join the SleepRhythm Unipd initiative. On joining it for the first time, they were asked to provide information on demographics, health‐related indices, sleep–wake timing, night sleep quality and diurnal sleepiness. They then received one of two sets of circadian hygiene advice: “A regular life” or “Bright days and dark nights” (figure 1 in Montagnese et al., 2022). Both included elements of the recommendations routinely provided to individuals with either a formal diagnosis and/or features of delayed sleep phase type (Abbott et al., 2015), which are common in adolescents and young adults. “A regular life” encouraged participants to regularise their habits. “Bright days and dark nights” encouraged participants to advance their sleep–wake, meals and exercise timing, and to maximise/minimise light exposure in the first/second part of the day, respectively (figure 1 in Montagnese et al., 2022). Every month after joining, participants were asked how easy it had been to comply (compliance was subjectively rated on a 0–10 visual‐analogue scale) with each piece of advice, and were provided with the same advice again. At every even month from joining, they were asked to complete demographic and sleep–wake information again. They were also asked how easy it had been to comply, and provided with the advice again. Date and time of completion of all procedures scheduled at time 0 (T0) were recorded. The month of entry into the study was recorded and corrected, where appropriate, for the presence of daylight saving time (DST) for use as a surrogate marker of light exposure. Study entry was also qualified as prior to, during (March 2020–September 2021) or after distance learning was instated/interrupted.

Electronic versions of the following questionnaires were administered, and scored according to pertinent Italian norms: the Sleep Timing Sleep Quality Screening (STSQS) questionnaire (Montagnese et al., 2009); the Pittsburgh Sleep Quality Index (PSQI; Buysse et al., 1989); the Epworth Sleepiness Scale (ESS; Johns, 1991); the Self‐Morningness/Eveningness (Self‐ME) questionnaire (Turco et al., 2015); the ultrashort version of the Munich ChronoType Questionnaire (μMCTQ; Ghotbi et al., 2020).

Descriptive results are expressed as mean ± SD or as count/percentage. Normality was tested for by the Shapiro–Wilk test. Differences between normally/non‐normally distributed variables were examined by the Student's *t*‐test/one‐way analysis of variance (ANOVA; post hoc: Scheffé test) and by the Mann–Whitney *U*‐test/Kruskal–Wallis ANOVA, respectively. Bonferroni correction was applied as appropriate. Factorial ANOVA was utilised for multiple grouping factors. A linear regression model was utilised to identify independent predictors of sleep–wake indices (amongst age, sex, chronotype, corrected month of entry and distance learning). Analyses were carried out with Statistica, version 14.0.0.15 (TIBCO, Palo Alto, CA, USA).

## 3 RESULTS

Between 28 October 2019 and 15 May 2023, 7766 students (4797 [61.8%] females, age 23 ± 5 years) joined the initiative; 3868 (49.8%) were assigned to the “Bright days and dark nights” and 3898 (50.2%) to the “A regular life” advice groups, and there were no significant differences between the two groups on any demographic, health‐related or sleep–wake variable. One‐thousand, four‐hundred and seven (18.2%) students reported having one or more diseases; amongst those who chose to qualify such diseases (free writing), 12 (0.15%) reported suffering from “insomnia”. One‐thousand, eight‐hundred and forty‐three (24%) students reported taking medication. As expected, there was significant overlap between students reporting diseases and those reporting being on medication (*χ* ^2^ = 1319, *p* < 0.0001). Significantly more female students reported having one/more diseases (20.3% versus 15.1%; *χ* ^2^ = 32, *p* < 0.0001). While reports of “insomnia” were rare, they were more common in males (Table S1).

Four‐thousand, six‐hundred and forty students (59.7%) had abnormal night sleep quality (PSQI score > 5), and 967 (12.4%) had excessive daytime sleepiness (ESS ≥ 11). In both instances, abnormal scores were more common in females (62.9 versus 54.5%, *χ* ^2^ = 54, *p* < 0.0001 and 14.6 versus 8.9%, *χ* ^2^ = 54, *p* < 0.0001, respectively). Midsleep on work/study and free days was 03:48 ± 01:12 and 05:06 ± 01:18, respectively, and in both instances it was significantly later in males (03:54 ± 01:18 versus 03:42 ± 01:12, *p* < 0.0001; 05:12 ± 01:24 versus 05:00 ± 01:18, *p* < 0.0001); social jet lag was 1.2 ± 1.2 hr, and it was significantly larger in males (1.3 ± 1.3 hr versus 1.2 ± 1.3 hr, *p* < 0.0001). Chronotype distribution, by sex, is presented in Table 1.

**TABLE 1:** Chronotype distribution, by sex.

	Extremely morning	Morning	Evening	Extremely evening	Total (*n*)
Males (*n*, %)	250 (8.4%)	934 (31.5%)	1181 (39. 8%)	604 (20.3%)	2969
Females (*n*, %)	557 (11.6%)	1602 (33.4%)	1833 (38.2%)	805 (16.8%)	4797
Total (*n*)	807	2536	3014	1409	7766

*Note*: *χ* ^2^ = 34, *p* < 0.0001.

As expected, extremely evening/evening students had significantly delayed sleep–wake habits compared with their extremely morning/morning counterparts, and they also had longer sleep latency, worse mood, worse night sleep quality, more daytime sleepiness, shorter sleep duration on work/study days, longer sleep duration on free days and more social jet lag (Table S2).

Three‐thousand, one‐hundred and eighty‐five (41.0%) students never filled in a follow‐up compliance or questionnaire. Depending on study entrance date, they had a period of time to do so that ranged between 2 and 43 months. The likelihood of never providing follow‐up data was higher in males (46.4% versus 37.6%; *χ* ^2^ = 58, *p* < 0.0001). Age and reported disease/insomnia were comparable in the two groups. The percentage of students allocated to the two types of advice was also comparable in the two groups. Students who never provided follow‐up information had later sleep–wake habits, more daytime sleepiness and worse night sleep quality compared with their counterparts who provided follow‐up information (Table 2). Chronotype distribution was also different, with a slight but significantly higher proportion of extremely evening/evening types amongst the early drop‐outs (*χ* ^2^ = 10, *p* < 0.05; Table S3). All the above analyses were repeated in the subgroup of students who had had a minimum of 12 months to provide follow‐up data (7016 in total, of whom 2804 [40%] were early drop‐outs), and the features of the early drop‐outs in this sample overlapped those of the larger one.

**TABLE 2:** Age, mood and sleep–wake features of students who did/did not engage in follow‐up.

	Follow‐up *n* = 4581	No follow‐up *n* = 3185
Age (years)	23.2 ± 5.4	23.3 ± 5.1
Mood (1–10)	5.9 ± 1.9	5.8 ± 1.8
ESS (total score, 0–24)	6.2 ± 3.5	6.4 ± 3.6**
PSQI (total score, 0–21)	6.7 ± 3.2	6.8 ± 3.1*
Sleep‐onset time (work/study days; clock time)	00:06 ± 01:12	00:12 ± 01:18***
Wake up time (work/study days; clock time)	07:30 ± 01:18	07:42 ± 01:36***
Midsleep (work/study days; clock time)	03:48 ± 01:12	03:54 ± 01:18***
Sleep duration (work/study days; hr)	7.4 ± 1.3	7.4 ± 1.4
Sleep‐onset time (free days; clock time)	00:54 ± 01:18	01:00 ± 01:30***
Wake up time (free days; clock time)	09:12 ± 01:30	09:18 ± 01:18*
Midsleep time (free days; clock time)	05:00 ± 01:12	05:12 ± 01:24***
Sleep duration (free days; hr)	8.4 ± 1.3	8.3 ± 1.4
Social jet lag (hr)	1.2 ± 1.1	1.2 ± 1.3

*Note*: *p < 0.05; **p < 0.01; ***p < 0.0001 (Bonferroni‐corrected for sleep–wake timing variables).

Abbreviations: ESS, Epworth Sleepiness Scale; PSQI, Pittsburgh Sleep Quality Index.

**FIGURE 1:**
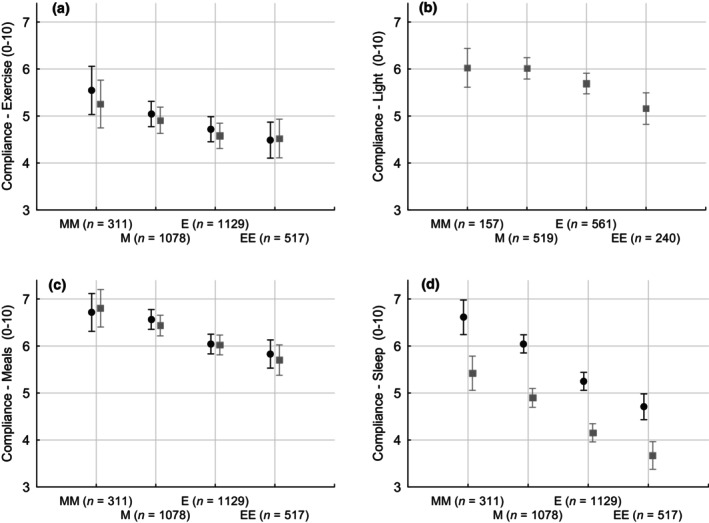
Self‐reported compliance (1–10 visual‐analogue scale, study time T1, that is, 1 month after joining; mean ± 95% confidence interval) to different types of advice (advice on exercise [a], light [b], meals [c], and sleep [d]) in the “Bright days and dark nights” (grey squares) and “A regular life” (black circles) groups, by chronotype (on the *x*‐axis). Chronotype for factorial ANOVA was significant in all instances (*p* < 0.0001; a–d). In the case of compliance to sleep advice (d), the advice group (“Bright days and dark nights” versus “A regular life”) was also significant (chronotype: *F* = 61, *p* < 0.0001; advice group: *F* = 132, *p* < 0.0001; interaction n.s.). Advice on light was provided only in the “Bright days and dark nights” group (b). E, evening; EE, extremely evening; M, morning; MM, extremely morning.

Three‐thousand and thirty‐five (39%) students completed the first compliance questionnaire, of which 1963 (64.7%) were females and 1072 (35.3%) males, with a significantly lower proportion of males compared with the initial T0 sample (*χ* ^2^ = 8, *p* < 0.01). Extremely evening/evening types were also significantly less prevalent compared with the T0 sample (*χ* ^2^ = 19, *p* < 0.001). Sleep quality was comparable in the two groups. Compliance was comparable in the “Bright days and dark nights” and “A regular life” groups for the advice on meal and exercise timing. Compliance for the advice on sleep timing was significantly lower in the “Bright days and dark nights” group (4.4 ± 2.4 versus 5.6 ± 2.4, *p* < 0.001). Compliance was significantly lower in extremely evening/evening chronotypes compared with their extremely morning/morning counterparts for all types of advice provided, with no effect of “Bright days and dark nights” versus “A regular life”, except for compliance with the advice on sleep timing. This was significantly lower in the “Bright days and dark nights” group, which advised sleep–wake timing anticipation rather than simply regular sleep–wake timing (chronotype: *F* = 61, *p* < 0.0001; advice group: *F* = 132, *p* < 0.0001; interaction n.s.; Figure 1).

**FIGURE 2:**
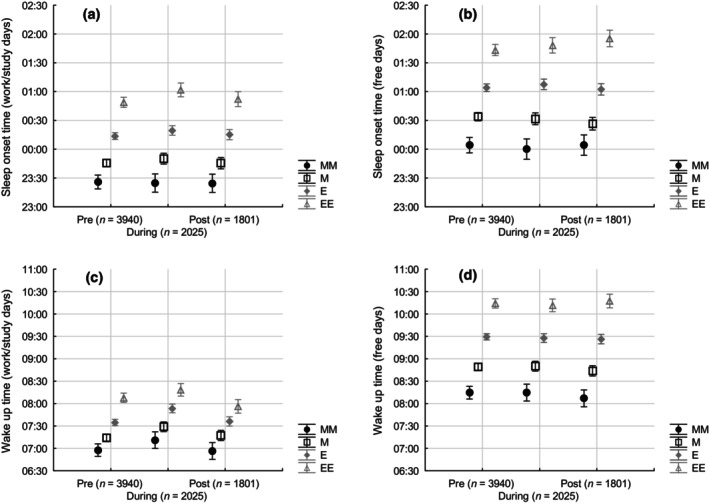
Sleep‐onset (mean ± 95% confidence interval; top panels, a, b) and wake up times (mean ± 95% confidence interval; bottom panels, c, d) on work/study days (left panels, a, c) and free days (right panels, b, d), by chronotype (extremely morning: black circles; morning: empty squares; evening: grey diamonds; extremely evening: empty triangles) and distance learning (on the *x*‐axis). On factorial ANOVA, chronotype was significant in all instances while distance learning was significant only on work/study days. The effect of distance learning was more obvious on wake up times on work/study days (c); here, the differences between chronotypes were less obvious than in all other instances, most likely as a result of the use of an alarm clock (c). (a) Chronotype: *F* = 344, *p* < 0.0001; distance learning: *F* = 3, *p* < 0.05; interaction n.s. (b) Chronotype: *F* = 385, *p* < 0.0001; distance learning n.s.; interaction n.s. (c) Chronotype: *F* = 132, *p* < 0.0001; distance learning: *F* = 19, *p* < 0.0001; interaction n.s. (d) Chronotype: *F* = 447, *p* < 0.0001; distance learning n.s.; interaction n.s. E, evening; EE, extremely evening; M, morning; MM, extremely morning.

Distance learning was associated with significant, large and temporary delays in sleep–wake timing, especially during work/study days. When the sleep timing effects of the combination of distance learning and chronotype were assessed, both were significant for work/study days, while only chronotype was significant for free days (Figure 2).

Models including age, sex, chronotype, month of entry into the study corrected for DST and distance learning as predictors of sleep–wake timing/quality indices showed that chronotype was a significant predictor for all sleep–wake timing/quality indices. All other predictors were also significant for most indices, with sleep quality and sleep offset on free days being generally—and not unexpectedly—insensitive to month of entry into the study and distance learning (Table S4).

## 4 DISCUSSION

This second, interim analysis of the circadian education initiative SleepRhythm Unipd confirmed the high prevalence of disturbed night sleep and social jetlag amongst students of Padova University, especially extremely evening/evening ones. Extremely evening/evening students also reported lower levels of compliance and were more likely to leave the initiative early. So were males compared with females. These observations are in line with the notion that an individual's ability to modify their natural inclination towards placing the main sleep episode in a certain period of the 24 hr, especially during adolescence and early adulthood, is limited (Hagenauer & Lee, 2012). It also suggests that circadian education for university students may be more effective for evening rather than extremely evening types, and extremely evening types may benefit, as an alternative, from delays in lecture timings and/or light therapy. Delayed or flexible lecture timings have been shown to be beneficial for sleep duration (Biller et al., 2022) and, in some instances, also for grades in high school (Dunster et al., 2018). Recently, daily schedules have been shown to influence sleep timing/duration also in undergraduate college students (Lu et al., 2023). Of interest, we were previously able to document how a short course of morning light administration/evening shades resulted in a significant decrease in sleep‐onset latency in a small group of university students (Montagnese et al., 2021). Adequately lit, dedicated communal areas may pleasantly serve the same purpose. Also, while the first interim analysis of the SleepRhythm Unipd initiative documented worse academic performance in evening/extremely evening students compared with their morning/extremely morning counterparts in all study subjects, it also showed a tendency of extremely evening students to perform better than evening ones (Montagnese et al., 2022). In view of the present data on early drop‐outs and compliance, it is possible to hypothesise that as their evening propensity increases, students may make less of an effort to comply with social timing constraints, study at their most productive time and ultimately perform better. Further, while in high school absenteeism is more likely to result in worse academic performance (Zerbini et al., 2017), this may not be the case in university. Finally, it is worth highlighting that in our study chronotype was defined on a previously validated single question (Turco et al., 2015), thus making it possible to imagine rapid and logistically easy screening of all students—for example on a voluntary basis on enrolment—in order to personalise strategies to combat the negative effects of misalignment on sleep duration and academic performance (Creswell et al., 2023). Within a productive society, sleep loss amongst young adults and impaired academic performance are costly in the medium‐ and long‐run (Hafner et al., 2017). By contrast, educational campaigns, light therapy and changes to university timetables may be more or less complex logistically but are cheap, especially once instated. It is therefore reasonable to imagine that any such intervention and especially their combination will be cost‐effective.

## AUTHOR CONTRIBUTIONS

**Gianluca Giusti:** Writing – original draft; formal analysis; data curation; visualization; investigation. **Esther D. Domenie:** Investigation; writing – original draft; visualization; formal analysis; data curation. **Lisa Zarantonello:** Methodology; formal analysis; project administration; data curation. **Chiara Mangini:** Visualization; writing – review and editing; data curation. **Paolo Ferrari:** Conceptualization; methodology; software; project administration; data curation; formal analysis. **Rodolfo Costa:** Conceptualization; investigation; funding acquisition; writing – review and editing; formal analysis; project administration; methodology; supervision. **Sara Montagnese:** Conceptualization; investigation; funding acquisition; writing – original draft; methodology; formal analysis; project administration; data curation; supervision; resources.

## CONFLICT OF INTEREST STATEMENT

PF is the Chief Executive Officer and owner of reMedia Srl., which received compensation for the development of the initiative full‐responsive website.

## Supporting information


**TABLE S1.** Reports of one/more diseases and insomnia, by sex.
**TABLE S2.** Age, mood and **s**leep–wake features (mean ± SD) at T0, by chronotype.
**TABLE S3.** Chronotype distribution, by presence of follow‐up data.
**TABLE S4.** Significance of a set of predictors on sleep–wake indices at T0.

## Data Availability

The data that support the findings of this study are available from the corresponding author upon reasonable request.
