# Supplementary material for: Personalising circadian hygiene educational initiatives aimed at university students—“He who has ears to hear, let him hear”
Source: J Sleep Res. 2024 Mar 14;33(6):e14194. doi: 10.1111/jsr.14194 (PMC11597023; doi:10.1111/jsr.14194)
Supplement: Supplementary file 1 — TABLE S1. Reports of one/more diseases and insomnia, by sex. TABLE S2. Age, mood and sleep–wake features (mean ± SD) at T0, by chronotype. TABLE S3. Chronotype distribution, by presence of follow‐up data. TABLE S4. Significance of a set of predictors on sleep–wake indices at T0. [file JSR-33-e14194-s001.docx]

**Supplemental Table 1.** Reports of one/more diseases and insomnia, by sex

|  | \| **No/negligible diseases** \| \| --- \| | \| **One/more diseases** \| \| --- \| | \| **Insomnia** \| \| --- \| | ***Total (n)*** |
| --- | --- | --- | --- | --- | --- | --- | --- |
| **Males (n, %)** | 2509 (84.85%) | 439 (14.85%) | 9 (0.30%) | 2957 |
| **Females (n, %)** | 3812 (79.70%) | 968 (20.24%) | 3 (0.06%) | 4783 |
| \| ***Total (n)*** \| \| --- \| | 6321 | 1407 | 12 | 7740* |

**26 (0.3%) of the 7766 students did not complete the relevant field*

*χ^2^=42, p<0.0001*

**Supplemental Table 2.** Age, mood and **s**leep-wake features (mean±SD) at T0, by chronotype

|  | **Extremely Morning**  **(n=807)** | **Morning (n=2536)** | **Evening (n=3014)** | **Extremely Evening (n=1409)** |
| --- | --- | --- | --- | --- |
| \| **Age (yrs)** \| \| --- \| | 24.4±7.4 | 23.2±5.1*^□□^* | 23.0±4.8*^••^* | 23.5±5.1**^,^*^$^* |
| \| **Mood (1-10)** \| \| --- \| | 6.3±1.9 | 6.1±1.8 | 5.7±1.8*^••,##^* | 5.4±2.0****^,††,$$$^* |
| \| **ESS (total score, 0-24)** \| \| --- \| | 6.0±3.4 | 6.0±3.4 | 6.4±3.5*^##^* | 6.5±3.8**^,††^* |
| \| **PSQI (total score, 0-21)** \| \| --- \| | 6.3±3.3 | 6.4±3.1 | 6.9±3.1*^••,##^* | 7.5±3.2****^,††,$$$^* |
| \| **Sleep onset time**  **(work/study days; clock time)** \| \| --- \| \|  \| | 23:24±01.06 | 23:48±01.06*^□□^* | 00:18±01:12*^••,##^* | 00:54±01.24****^,††,$$$^* |
| \| **Wake up time**  **(work/study days; clock time)** \| \| --- \| | 07:00±01:12 | 07:18±01:12*^□^* | 07:42±01:24*^••,##^* | 08:06±01:42****^,††,$$^* |
| \| **Midsleep**  **(work/study days; clock time)** \| \| --- \| | 03:12±01:00 | 03:30±01:00*^□□^* | 04:00±01:18*^••,##^* | 04:30±01.18****^,††,$$$^* |
| \| **Sleep duration**  **(work/study days; hours)** \| \| --- \| | 7.6±1.2 | 7.5±1.2 | 7.4±1.4 | 7.2±1.5**^,†,$^* |
| \| **Sleep onset time**  **(free days; clock time)** \| \| --- \| | 00:06±01:18 | 00:30±01:18*^□□^* | 01:06±01:24*^••,##^* | 01:48±01:24****^,††,$$$^* |
| \| **Wake up time**  **(free days; clock time)** \| \| --- \| | 08:12±01:18 | 08:48±01.18*^□□^* | 09:30±01:24*^••,##^* | 10:12±01:18****^,††,$$$^* |
| \| **Midsleep time**  **(free days; clock time)** \| \| --- \| | 04:06±01:12 | 04:42±01:06*^□□^* | 05:18±01:12*^••,##^* | 06:00±01:18****^,††,$$$^* |
| \| **Sleep duration**  **(free days; hours)** \| \| --- \| | 8.2±1.3 | 8.3±1.3 | 8.4±1.4 | 8.4±1.4*^†^* |
| \| **Social jet lag (hours)** \| \| --- \| | 1.0±1.1 | 1.1±1.0 | 1.3±1.3*^•,#^* | 1.5±1.3****^,††,$$$^* |

*ESS, Epworth Sleepiness Scale; PSQI, Pittsburgh Sleep Quality Index*

*EE vs MM *p<0.05, **p<0.01, ***p<0.001; EE vs M ^†^p<0.05, ^††^ p<0.001; EE vs E ^$^p<0.05,  ^$$^p<0.01,  ^$$$^p<0.001
E vs MM ^•^p<0.05,  ^••^p<0.001; E vs M  ^#^p<0.05; ^##^p<0.001
M vs MM  ^□^p<0.05, ^□□^p<0.001*

**Supplemental Table 3.** Chronotype distribution, by presence of follow up data

|  | **Extremely morning** | **Morning** | **Evening** | **Extremely evening** | ***Total (n)*** |
| --- | --- | --- | --- | --- | --- |
| **Follow up** | 463 (10.1%) | 1560 (34.1%) | 1741 (38.0%) | 817 (17.8%) | 4581 |
| **No follow up** | 344 (10.8%) | 976 (30.6%) | 1273 (40.0%) | 592 (18.6%) | 3185 |
| \| ***Total (n)*** \| \| --- \| | 807 | 2536 | 3014 | 1409 | 7766 |

*χ^2^=10, p<0.05*

**Supplemental Table 4.** Significance and weights [the larger partial eta-squared (η_p_^2^) and the adjusted R squared, the larger the weight of the variable, i.e. the proportion of the variability in the dependent variables explained by the effect] of different predictors on a set of sleep-wake indices at T0

|  | **Age** | **Sex** | **Month of entry (corrected for DST)** | **Chronotype** | **Distance Learning** |
| --- | --- | --- | --- | --- | --- |
| \| **Sleep onset time (work/study days)** \| \| --- \| | p=0.03 | p<0.0001 | p=0.2 | p<0.0001 | p<0.01 |
|  | F=4.9 | F=34.7 | F=1.4 | F=360.5 | F=6.0 |
|  | ηp^2^=0.88 | ηp^2^=0.0006 | ηp^2^=0.004 | ηp^2^=0.002 | ηp^2^=0.122 |
|  | R^2^=0.01 | R^2^=0.01 | R^2^=0.51/0.74/0.79/0.48/0.83/0.83/0.73/0.47/0.88/0.80 | R^2^=0.42/0.28/0.27 | R^2^=0.36/0.23 |
| **Wake up time (work/study days)** | p<0.0001 | p<0.01 | p<0.0001 | p<0.0001 | p<0.0001 |
|  | F=71.8 | F=7.7 | F=3.7 | F=147.2 | F=32.8 |
|  | ηp^2^=0.009 | ηp^2^=0.001 | ηp^2^=0.005 | ηp^2^=0.05 | ηp^2^=0.008 |
|  | R^2^=0.01 | R^2^=0.01 | R^2^=0.51/0.74/0.79/0.48/0.83/0.83/0.73/0.47/0.88/0.80 | R^2^=0.42/0.28/0.27 | R^2^=0.36/0.23 |
| **Midsleep (work/study days)** | p<0.0001 | p<0.0001 | p<0.01 | p<0.0001 | p<0.0001 |
|  | F=56.5 | F=18.9 | F=2.5 | F=288.9 | F=18.6 |
|  | ηp^2^=0.007 | ηp^2^=0.002 | ηp^2^=0.003 | ηp^2^=0.1 | ηp^2^=0.005 |
|  | R^2^=0.01 | R^2^=0.01 | R^2^=0.51/0.74/0.79/0.48/0.83/0.83/0.73/0.47/0.88/0.80 | R^2^=0.42/0.28/0.27 | R^2^=0.36/0.23 |
| **Sleep duration (work/study days)** | p<0.0001 | p=0.01 | p<0.001 | p<0.0001 | p<0.0001 |
|  | F=52.2 | F=6.1 | F=3.2 | F=18.9 | F=16.2 |
|  | ηp^2^=0.007 | ηp^2^=0.0008 | ηp^2^=0.004 | ηp^2^=0.007 | ηp^2^=0.004 |
|  | R^2^=0.01 | R^2^=0.01 | R^2^=0.51/0.74/0.79/0.48/0.83/0.83/0.73/0.47/0.88/0.80 | R^2^=0.42/0.28/0.27 | R^2^=0.36/0.23 |
| \| **Sleep onset time**  **(free days)** \| \| --- \| | p<0.0001 | p<0.0001 | p=0.8 | p<0.0001 | p=0.8 |
|  | F=64.1 | F=64.8 | F=0.6 | F=394.1 | F=0.2 |
|  | ηp^2^=0.008 | ηp^2^=0.008 | ηp^2^=0.0008 | ηp^2^=0.13 | ηp^2^=0.00005 |
|  | R^2^=0.01 | R^2^=0.01 | R^2^=0.51/0.74/0.79/0.48/0.83/0.83/0.73/0.47/0.88/0.80 | R^2^=0.42/0.28/0.27 | R^2^=0.36/0.23 |
| \| **Wake up time**  **(free days)** \| \| --- \| | p<0.0001 | p=0.04 | p=0.1 | p<0.0001 | p=0.3 |
|  | F=320.8 | F=4.2 | F=1.6 | F=491.4 | F=1.2 |
|  | ηp^2^=0.04 | ηp^2^=0.0005 | ηp^2^=0.002 | ηp^2^=0.2 | ηp^2^=0.003 |
|  | R^2^=0.01 | R^2^=0.01 | R^2^=0.51/0.74/0.79/0.48/0.83/0.83/0.73/0.47/0.88/0.80 | R^2^=0.42/0.28/0.27 | R^2^=0.36/0.23 |
| **Midsleep time**  **(free days)** | p<0.0001 | p<0.0001 | p<0.01 | p<0.0001 | p=0.2 |
|  | F=227.4 | F=33.0 | F=0.7 | F=588.8 | F=0.4 |
|  | ηp^2^=0.03 | ηp^2^=0.004 | ηp^2^=0.0008 | ηp^2^=0.2 | ηp^2^=0.00009 |
|  | R^2^=0.01 | R^2^=0.01 | R^2^=0.51/0.74/0.79/0.48/0.83/0.83/0.73/0.47/0.88/0.80 | R^2^=0.42/0.28/0.27 | R^2^=0.36/0.23 |
| **Sleep duration**  **(free days)** | p<0.0001 | p<0.0001 | p=0.7 | p<0.0001 | p=0.7 |
|  | F=108.9 | F=32.5 | F=2.5 | F=11.0 | F=1.8 |
|  | ηp^2^=0.01 | ηp^2^=0.004 | ηp^2^=0.003 | ηp^2^=0.004 | ηp^2^=0.0004 |
|  | R^2^=0.01 | R^2^=0.01 | R^2^=0.51/0.74/0.79/0.48/0.83/0.83/0.73/0.47/0.88/0.80 | 0.42/0.28/0.27 | 0.36/0.23 |
| **Social jet lag** | p<0.0001 | p=0.2 | p<0.01 | p<0.0001 | p<0.0001 |
|  | F=55.4 | F=2.0 | F=2.4 | F=53.5 | F=15.7 |
|  | ηp^2^=0.007 | ηp^2^=0.0002 | ηp^2^=0.003 | ηp^2^=0.02 | ηp^2^=0.004 |
|  | R^2^=0.01 | R^2^=0.01 | R^2^=0.51/0.74/0.79/0.48/0.83/0.83/0.73/0.47/0.88/0.80 | R^2^=0.42/0.28/0.27 | R^2^=0.36/0.23 |
| \| **ESS (total score)** \| \| --- \| | p=0.8 | p<0.0001 | p=0.03 | p<0.0001 | p<0.0001 |
|  | F=0.1 | F=143.6 | F=1.9 | F=12.4 | F=10.6 |
|  | ηp^2^=0.05 | ηp^2^<0.0001 | ηp^2^=0.02 | ηp^2^=0.002 | ηp^2^=0.005 |
|  | R^2^=0.01 | R^2^=0.01 | R^2^=0.51/0.74/0.79/0.48/0.83/0.83/0.73/0.47/0.88/0.80 | R^2^=0.42/0.28/0.27 | R^2^=0.36/0.23 |
| \| **PSQI (total score)** \| \| --- \| | p<0.0001 | p<0.0001 | p=0.6 | p<0.0001 | p<0.001 |
|  | F=69.8 | F=143.0 | F=0.8 | F=54.7 | F=8.3 |
|  | ηp^2^=0.009 | ηp^2^=0.02 | ηp^2^=0.001 | ηp^2^=0.02 | ηp^2^=0.002 |
|  | R^2^=0.01 | R^2^=0.01 | R^2^=0.51/0.74/0.79/0.48/0.83/0.83/0.73/0.47/0.88/0.80 | R^2^=0.42/0.28/0.27 | R^2^=0.36/0.23 |

*ESS, Epworth Sleepiness Scale; PSQI, Pittsburgh Sleep Quality Index; DST, Daylight Saving Time*
